# Burlap and buddies: the effects of social enrichment (preweaning mixing) and object enrichment (burlap) on piglet behavior and welfare in the postweaning environment

**DOI:** 10.1093/tas/txae057

**Published:** 2024-04-09

**Authors:** Ashlyn Scott, Arielle Le Heiget, Reyna Stefanson, Jamie Ahloy-Dallaire, Meagan King

**Affiliations:** Department of Animal Science, University of Manitoba, Winnipeg, MB R3T 2N2, Canada; Department of Animal Science, University of Manitoba, Winnipeg, MB R3T 2N2, Canada; Department of Animal Science, University of Manitoba, Winnipeg, MB R3T 2N2, Canada; Department of Animal Science, University of Manitoba, Winnipeg, MB R3T 2N2, Canada; Département des sciences animales, Université Laval, Québec, QC G1V 0A6, Canada; Department of Animal Science, University of Manitoba, Winnipeg, MB R3T 2N2, Canada

**Keywords:** behavior, enrichment, piglet, postweaning, welfare

## Abstract

The process of weaning piglets in commercial swine operations subjects them to numerous abrupt and stressful changes often resulting in negative welfare consequences. The objective was to study the postweaning effects of early-life (1 to 3 d of age) preweaning socialization in multi-litter groups as well as object enrichment (burlap sheet) in the pre- and postweaning environment by comparing six treatments that combined mixing of one vs. two vs. four litters mixed preweaning with and without burlap provision. An ANOVA linear model was run on all normal data, expressed per experimental unit (and behavior data were averaged over time), while non-normal data were analyzed using the Kruskal–Wallis test. Non-enriched groups of piglets were observed manipulating pen objects more often than the enriched groups (*P *= 0.005). Biting behaviors, including the chewing of ears and tails of pen-mates but excluding fighting, were observed the least in groups of pigs of four litters mixed preweaning, while piglets that were not mixed preweaning were observed biting the most (*P *= 0.03). Piglets who were not mixed preweaning also manipulated the burlap more frequently than the piglets from groups of 4 litters mixed preweaning (*P *= 0.02). Biting (*P *< 0.001) and displacements (*P *= 0.03) and fighting (*P *= 0.002) throughout the pen were observed less in the enriched groups. There were fewer lesions per pig in the enriched groups vs. non-enriched groups initially (*P *= 0.07) and 1 wk after weaning (*P *= 0.10). Furthermore, pigs mixed in groups of four litters preweaning also tended to have lower lesion scores (*P *= 0.07) 1-wk postweaning compared to the other treatments. However, there were no differences between treatments in the proportion of piglets resting, eating/drinking, being active, or using the burlap, or for the observed frequency of displacements at the feeder, social behaviors, or belly nosing (*P *> 0.10). Overall, social enrichment encourages socialization with unfamiliar conspecifics at a younger age while object enrichment allows pigs to redirect their attention toward objects such as burlap. Both may improve pig behavior and welfare after weaning.

## Teaser

Both social and object enrichment were found to benefit piglets at weaning. Depending on the suitability of each enrichment type to the operation as well as individual producers’ preferences, social and/or object enrichment are feasible to implement in commercial operations.

## Introduction

The weaning process in multistage commercial swine operations is often the first major disruption in a piglet’s life; it is also more abrupt and at an earlier age than would be observed naturally (Salazar et al., 2018). During the weaning transition, piglets may be separated from their littermates and must form a new social hierarchy with new pen-mates. The piglets must also adapt to a new environment and new feed, as well as being without their sow. They experience increased stress, changes to their physical and social environments, and the additional stress of handling and potentially being transported. Moving piglets to a nursery facility usually requires being transported by livestock trailer in large groups of unfamiliar piglets. If transport off-site is not required, the moment piglets are regrouped in the nursery would be when they first meet unfamiliar piglets. As a result, the weaning transition can have detrimental effects on piglets’ welfare, health, and production parameters around weaning which may influence future performance and welfare (Ko et al., 2020). Therefore, producers are continuously working towards reducing the impact of stress associated with the weaning transition on their piglets. Effective methods to reduce piglet stress around weaning should focus on encouraging natural behaviors while also considering associated labor and costs, especially to be feasible in large-scale, commercial operations.

The establishment of social hierarchies often results in heightened aggression and agonistic behaviors among groups of unfamiliar pigs (i.e., newly weaned pigs; Ledergerber et al., 2015). Weaning is commonly associated with a growth check for piglets (Schmitt et al., 2020). Ideally, piglets would experience a smooth transition from their sow’s milk to solid feed, from the farrowing pen to a nursery pen, and to a new social group with a minimal negative impact on their health, performance, and behavior. It has been shown that allowing piglets to socialize with other litters preweaning improves a piglet’s adaptability once weaned, allowing hierarchies to be established faster when regrouped (D’Eath, 2005; Hessel et al., 2006). What has not yet been well documented is if varying the number of litters mixed before weaning has an impact on piglet behavior after weaning. Mixing groups of two to three litters before weaning has been previously studied and shown to be advantageous in terms of piglet behavior (less aggression) and welfare around weaning (Hessel et al., 2006; Parratt et al., 2006; Salazar et al., 2018; Van Kerschaver et al., 2021). Further research is needed to evaluate whether mixing young pigs in groups greater than three litters is beneficial for the adaptability of piglets to subsequent mixing. In a study evaluating the impact of pig group size postweaning (20 vs. 80 × 30-kg growing pigs) on aggressive behavior, pigs that were housed in the larger groups displayed less aggression toward unfamiliar pigs when introduced in pairs to a pair of unknown pigs 6 wk after being weaned (Turner et al., 2001). Therefore, preweaning socialization may spread out stressors for young pigs, as well as give them an opportunity to develop transferable social skills that can continue to be used at future mixing events, such as at weaning, thereby improving piglet behavior and welfare (D’Eath, 2005; Morgan et al., 2014; Ko et al., 2020; Fels et al., 2021).

It is also common for pig production facilities to keep the animals’ environment simple (barren) to allow ease of cleaning between groups of pigs, thereby reducing biosecurity risks. Barren environments can predispose pigs to perform more agonistic behaviors such as biting of ears and tails, and belly nosing, especially when stressed. Providing piglets with access to enrichment may provide a positive outlet for stress and reduce the frequency of undesirable behaviors during weaning; burlap, specifically, has been shown to have the potential to benefit pigs raised in commercial operations (Fynn et al., 2021). Burlap is an ideal form of object enrichment for commercial swine production because it is cost-effective, poses little risk to the manure system when provided to young pigs, and poses no biosecurity risk if a new sheet can be used for each batch. Alternative types of enrichment such as substrates (straw) or rubber toys have a higher risk of damaging manure systems and require additional cleaning or sanitization practices to be implemented, respectively. Additionally, Schmitt et al. (2020) found that piglets preferred manipulating (shaking and biting) burlap over other enrichment (bamboo). It has been hypothesized that pigs prefer enrichments that they can destroy since it contributes to the novelty of the enrichment (NFACC, 2021).

Although it is not until the finishing stage when the negative effects of biting behaviors among pigs become apparent in victims, biting behaviors typically develop at a young age in pigs (Hakansson and Bolhuis 2021). Therefore, providing burlap sheets as enrichment as early as possible in a pig’s life may redirect biting behaviors from their pen-mates to the burlap sheet providing a positive outlet for the development of their natural chewing behaviors, improving the welfare of both the biter and the victim. To facilitate the transition for weaned pigs, studies have evaluated providing enrichment in the postweaning environment (Ledergerber et al., 2015) while few have evaluated its impact when provided in both pre- and postweaning environments. Oostindjer et al. (2011) found that postweaning enrichment, as compared to preweaning enrichment, had the strongest effect on postweaning behavior; piglets with enrichment postweaning performed more exploratory behaviors and fewer agonistic behaviors such as belly nosing and biting/chewing of pen-mates (Oostindjer et al., 2011).

A recent study looked at both object enrichment and preweaning socialization as a strategy to reduce regrouping stress and aggression, but those authors combined object and social enrichment in one treatment and compared it to a completely barren environment (Ko et al., 2020). Therefore, it is currently unknown whether social or object enrichment is more beneficial, as well as whether the combined effects of early mixing and environmental enrichment are additive or synergistic.

The objectives of this study were to determine the postweaning effects of: (1) providing piglets with a burlap sheet (object enrichment) in both their pre- and postweaning environment, (2) mixing litters early in life (social enrichment) at different levels of mixing (two or four litters mixed preweaning), and (3) a combination of both object and social enrichment.

## Materials and Methods

This study was conducted during the summer of 2022 (June to August) at a conventional nursery barn in South-East Manitoba, Canada. All experimental procedures were approved by the Animal Care Committees of the Research Ethics Board (Protocol Reference Number: F21-022, AC11708) at the University of Manitoba, Fort Garry campus. Experimental factors (social and object enrichment) did not interfere with routine pig care and management procedures, which were conducted by barn staff.

### Animals, Housing, and Experimental Design

The nursery facility consisted of four barns; each barn had four separate rooms, designated as blocks in our study. Trial pigs were housed in each of the four rooms in one barn per replicate. At weaning (nursery day 0), pigs were moved by livestock trailer to the off-site nursery barn (approximately 30 min travel time). All piglets originated from the same sow barn. Upon arrival at the nursery, in line with current standard practice at the barn, pigs were sorted by treatment and sex, into groups with an average of 22.3 pigs per pen (min. 15 pigs; max. 27 pigs) with comparable floor space allowance (per pig) across treatments. All pigs were housed in 10  × 6 ft (3.05  × 1.83 m) pens with two wall-mounted water nipples, slatted floors, and a five-space-free-access dry feeder. The animals were on a wheat/ corn/ soybean meal crumble diet. The lights in the nursery rooms were on 8-h timers and temperature was monitored continuously using hanging probs connected to the barn’s temperature control system with the minimum and maximum temperature readings manually recorded daily by barn staff on a per-room basis.

The estimated minimum sample size of the number of experimental units needed to detect treatment differences was calculated based on previous studies with sample sizes of 32, 12 and 20, and 27 (Camerlink et al., 2018; Salazar et al., 2018; Van Kerschaver et al., 2021), respectively. Using treatment means and standard deviation, or to detect a 10% difference among treatments, the sample size needed was calculated to range from 12 to 40 per treatment for behavior and 2 to 14 per treatment for lesions with 80% power and an alpha of 0.05 (Hessel et al., 2006; Morgan et al., 2014; Ko et al., 2020).

There were two replicates run in the nursery. One of the two replicates was split into two weaning groups due to the weaning dates being different between farrowing rooms.

To assess the effects of mixing litters preweaning and burlap provision on piglet behavior and welfare after weaning, in a 3 × 2 factorial experiment, litters were assigned to one of the six treatments in the sow barn: 1 litter, not enriched (1N; *n* = 14), 1 litter, enriched (1E; *n* = 15), 2 litters, not enriched (2N; *n* = 16), 2 litters, enriched (2E; *n* = 15), 4 litters, not enriched (4N; *n* = 15), 4 litters, and enriched (4E; *n* = 14). Treatments were randomly assigned and applied in the sow barn by removing the plastic divider that separated the farrowing pens and hanging burlap at the back of the pen when applicable. Treatments were carried over to the nursery stage when the pigs were weaned at an average age of 22  ± 4 d. Therefore, the piglets remained on the same treatment before and after weaning.

Prior to weaning, piglets were painted according to treatment (six different colors) to facilitate the identification and sorting at the nursery. Due to the logistics of the weaning process and transportation between barns, it was not possible to separate the piglets by the farrowing room when weaned. For a more detailed description of the experimental design and preweaning results, refer to the companion manuscript (Scott et al., 2024).

In the nursery, piglets were regrouped into pens (experimental units) with others from the same treatment. There were 10 to 12 pens on trial per room (*n* = 89 experimental units total). The allocation of treatments was randomized within rooms using a random number generator and each treatment was present in each room (block).

The trial began on nursery day 0, after the pigs were sorted into the pens where the treatments were applied. Preweaning mixing was previously applied in the sow barn where two or four neighboring crates were allowed to socialize or kept as a single crate (current standard practice), based on treatment. Additional environmental enrichment was provided in the sow barn and the nursery to the enriched treatment groups, as a burlap sheet. Two 30.5 cm wide sheets of burlap were secured using C-clamps on the side of the nursery pen for the enriched treatment groups and was an appropriate length to just reach the ground for ease of access for the piglets. The two 30.5 cm wide sheets of burlap were provided in each enriched pen based on calculations to provide enough space for approximately six pigs (~25% of the pigs in each pen) to use it simultaneously. After 1 wk in the nursery, at the conclusion of the study, the burlap was removed to preserve the integrity of the manure system.

### Behavioral Observations

On nursery days 1, 6, and 8 ± 1 d, two types of behavioral observations were performed: (1) scan observations aimed to capture the state behaviors of the pigs (eating/drinking, resting, active, and using burlap), and (2) continuous observations aimed to capture the event behaviors among weaned pigs throughout the pen, at the feeder/waterers, and at the burlap (fighting, biting, socializing, displacements, etc.; Table 1). Scan behavior observations were made by counting the number of piglets performing each of the listed behaviors: eating/drinking, resting, active, using burlap, at the start of each of the four observation times on each observation day. Continuous behavior observations used one-zero sampling, recording two consecutive 30-s observation periods. First, behavioral scans were recorded, followed by the two 30-s intervals. All pens per room were observed before moving to the next room, and all treatments in all rooms were observed four times per observation day (two in AM, two in PM). Four trained observers with a moderate or higher, interobserver reliability score for each behavior performed in-person observations.

**Table 1.** Ethogram of postweaning pig behaviors assessed to compare the effects of object enrichment (burlap provision) and early-life mixing of litters (social enrichment)

Behavior	Description	Behavior, sampling type	References
*Aggressive*			
Fighting	Physical encounters between at least two pigs including head-to-head fights, biting another pig, as well as pushing or knocking another pig with the head causing one pig to retreat/withdraw or both pigs engaging in aggression. May or may not include vocalizations.	Event, Continuous	Ledergerber et al. (2015) Ji et al. (2021)
Tail/ear biting	Pigs engaged in oral manipulation of pen mate’s tail or ear, may or may not result in wounds.	Event, Continuous	Hakansson and Bolhuis (2021)
Belly nosing	Piglet engaged in rhythmic nudging of another piglet’s abdomen (belly) with their nose; at least three nudges in a row.	Event, Continuous	Widowski et al. (2008)
Displacement	Physical contact between pigs results in one pig losing control over teat/object or needing to move or being pushed out of the way.	Event, Continuous	
*Investigative*			
Socializing	Pigs engaged in actions that did not cause the recipient to react negatively. Ex: nudging: snout of piglet is used to gently touch another pig’s body, not belly nosing.	Event, Continuous	Morgan et al. (2014) Yang et al. (2018)
Pen facilities	Nosing, licking, or chewing any object that is part of the pen (e.g., feeder), but excluding the enrichment object. Excluding any feeding behavior.	Event, Continuous	Yang et al. (2018)
Burlap	Manipulating (M) or investigating (I) the enrichment (burlap placed in the pen by the researcher) with mouth or snout, resulting in visible movement of the target (M) or sniffing or staring at enrichment within 1 foot of it (I).	Event, Continuous	Yang et al. (2018)
*General*			
Eating/drinking	Interacting with feed or waterers; Pig is standing at the trough with head down; the head can either be in the trough or in front of the trough when pigs eat feed.	State, Scan	Morgan et al. (2014)
Active	Locomotion (running or walking), Climbing (piglet uses its feet to elevate itself onto the body of another piglet or feeder), Standing (all four legs supporting the body with no ambulation or touching anything with their nose or mouth).	State, Scan	Yang et al. (2018) Morgan et al. (2014)
Resting	Lying (whole length of body on the floor or on other pigs, i.e., not supported by their legs) or sitting (Hind quarters on the floor, front legs supporting body).	State, Scan	Morgan et al. (2014) Ledergerber et al. (2015)

### Lesion Scoring

Piglet lesions were scored twice in the nursery using descriptions provided by Turpin et al. (2017) which assessed the severity of scratches (red marks and scabs) on the piglets’ ears, tail, and body. Lesions were scored initially in the nursery within 24 h postweaning, after being sorted into their respective pens, and on nursery day 7 ± 1 d postweaning to get a final score. Lesions were scored by one observer. The number of lesions present in each pen of pigs was counted and then the number of lesions (expressed per pig to standardize across different group sizes) was calculated for each experimental unit by taking the total number of (body lesions + tail lesions + ear lesions)/total number of piglets in that pen. The change in the number of lesions per pig was calculated by taking the difference between the number of lesions present initially and the number of lesions present on the final scoring day.

### Performance

To preserve the integrity of the weaning process, initial pen weights were not measured once the pigs were initially regrouped in the nursery to not further disturb the pigs and potentially cause additional stress to the weaning process. Furthermore, weighing each group of pigs in the nursery after weaning is not conducive to a “typical wean” and may have influenced piglet behavior. The objective was to mimic typical commercial settings as much as possible. Average daily gain, feed intake, and feed conversion efficiency could not be calculated since no feed intake data could be collected.

Mortality data were collected by barn staff on a per-pen basis. Only one piglet died on trial; therefore, mortality data were not included in this analysis. Furthermore, a sick pen was used for any piglets that were unfit to remain housed with other healthy individuals or if they needed medical intervention. During our trial, a total of six pigs were relocated to the sick pen: four unthrifty pigs, one lame pig, and one herniated pig.

### Statistical Analyses

All data were expressed per experimental unit and averaged over time. An ANOVA linear model was run on all data that met the normality criteria using the Shapiro–Wilks test on the residuals of the model and the Bartlett test. Significant differences were declared at *P *< 0.05 and tendencies were declared at 0.05 < *P *< 0.1. If significant differences or tendencies were found, an LSD-post hoc test was used to run pair-wise comparisons between treatments. Block (room) and replicate were included in the model to account for block and replicate effects. Treatment factors (number of litters mixed and enrichment) were included to assess any interaction effects. Non-normal conforming data were analyzed using the Kruskal–Wallis test.

## Results and Discussion

### Piglet Behavior

#### Effects of preweaning mixing (social enrichment)

Biting behaviors were influenced by preweaning mixing in our study (*P *= 0.003; Table 2); piglets that were mixed in groups of four litters preweaning were observed biting the least while piglets that were not mixed preweaning were observed biting the most and piglets that were mixed in groups of only two litters preweaning performed an intermediate amount of biting that did not differ from other levels of mixing.

**Table 2.** Percentage of observations where piglet behaviors were observed across six treatments^1^ to compare the effects of object enrichment (burlap provision) and preweaning mixing of litters (social enrichment)

Behavior	1E	1N	2E	2N	4E	4N	Mix factor *P*-value^2^	Burlap factor *P*-value^2^	Interaction effect *P*-value^2^
Investigating Burlap	5.6 ± 5.8	N/A	5.6 ± 4.9	N/A	6.0 ± 4.8	N/A	0.85	N/A	N/A
Manipulating Burlap	37.8 ± 18.5	N/A	37.2 ± 13.1	N/A	23.8 ± 14.3	N/A	**0.005**	N/A	N/A
Belly nosing	12.5 ± 11.6	15.9 ± 14.8	11.1 ± 13.7	18.1 ± 12.1	13.4 ± 12.2	20.4 ± 19.5	0.91	0.49	0.91
Biting	29.9 ± 11.7	47.2 ± 16.6	27.1 ± 8.3	39.3 ± 14.2	15.8 ± 12.1	37.9 ± 16.1	**0.003**	**<0.001**	0.16
Displacement (pen)	15.0 ± 9.6	22.6 ± 9.4	13.3 ± 7.2	19.1 ± 7.8	10.1 ± 7.2	21.0 ± 12.0	0.24	**0.03**	0.43
Displacement (feeder)	6.0 ± 5.3	9.1 ± 7.2	7.5 ± 5.5	6.4 ± 5.7	4.6 ± 5.0	6.4 ± 6.5	0.38	0.19	0.39
Fighting (pen)	9.6 ± 6.7	19.2 ± 12.3	9.4 ± 7.5	13.4 ± 7.3	6.7 ± 7.9	11.7 ± 6.9	0.45	**0.002**	0.58
Pen object manipulation	19.0 ± 11.5	32.9 ± 13.6	24.6 ± 13.6	32.0 ± 16.9	20.5 ± 11.8	26.3 ± 15.5	0.43	**0.005**	0.43
Socializing	28.6 ± 13.6	32.1 ± 11.0	31.0 ± 11.5	30.6 ± 11.0	27.1 ± 13.5	27.8 ± 13.5	0.60	0.40	0.65

^1^Expressed as mean ± SD per experimental unit, 1 1N (*n* = 14 × 1 litter, not enriched), 1E (*n* = 15 × 1 litter, enriched), 2N (*n* = 16 × 2 litters, not enriched), 2E (*n* = 15 × 2 litters, enriched), 4N (*n* = 15 × 4 litters, not enriched), and 4E (*n* = 14 × 4 litters, enriched). ^2^ Statistical significance declared at a P-value of 0.05 (denoted by bold text).

Mixing piglets preweaning has been previously shown to reduce observed aggressive behaviors associated with establishing a hierarchy within groups (Hessel et al., 2006). In previous studies, mixing piglets before weaning reduced aggressive behaviors after weaning, presumably because piglets were more quickly establishing a new, stable social hierarchy with the social skills previously established (Hessel et al., 2006; Ledergerber et al., 2015; Fels et al., 2021; Van Kerschaver et al., 2021). Our results and those of Camerlink et al. (2018) indicate that preweaning mixing has little effect on agonistic piglet behaviors (other than biting) postweaning.

Although the observed frequency of the burlap being investigated was not impacted by the mixing factor (*P* > 0.10), the actual manipulation of the burlap was impacted by the mixing factor (*P* = 0.005; Table 2). Burlap manipulation was observed most frequently in the groups of piglets from single litters while piglets from groups of four litters were observed manipulating the burlap the least (*P *= 0.02) which could indicate higher stress for piglets regrouped from single litters preweaning.

It could be speculated that burlap use indicates the level of stress the piglets experienced as a result of weaning because providing enrichment to piglets around weaning has previously been shown to be an effective strategy for reducing stress (Ledergerber et al., 2015). In the current study, burlap use was observed more in the single litter groups compared to those that were mixed in groups of four litters preweaning; this could indicate that piglets in the single litter groups were using it as an outlet for their stress associated with being mixed with unfamiliar pigs, not having previous opportunities to socialize. Piglets from the larger mixed groups may have developed social skills that they could then apply in the postweaning environment, or they were familiar with more individuals, resulting in reduced stress due to mixing at weaning and reducing the development of agonistic behaviors, such as chewing and biting. Others also found that mixing piglets preweaning provided piglets with an opportunity to develop social skills preweaning thereby reducing the incidence of agonistic behaviors and improving welfare (D’Eath, 2005; Morgan et al., 2014; Ko et al., 2020; Fels et al., 2021). Given the fact that space allowance (or stocking density) did not differ significantly across treatments (*P* > 0.10), observed differences in behavior are more likely to be attributed to treatment.

#### Effects of burlap provision (object enrichment)

Our results indicate that having access to enrichment reduced the frequency of agonistic behaviors observed between piglets. Biting (*P* < 0.001) and displacements (*P* = 0.03) throughout the pen were significantly lower in the enriched groups compared to the non-enriched groups (Table 2). Furthermore, piglets in the non-enriched groups were observed fighting throughout the pen more frequently than enriched groups (*P* = 0.002). These results indicate that piglets were redirecting their energy and aggression toward the burlap, which is further supported by previously published studies (D’Eath, 2005; Ledergerber et al., 2015).

Additionally, non-enriched groups of piglets were observed manipulating pen objects more often than the enriched groups (*P* = 0.005; Table 2). This could indicate that, if piglets are provided with a safe, soft, and sanitary enrichment object that encourages their natural oral manipulation behaviors (NFACC, 2021), they will be less likely to manipulate other objects around the pen (feeders, waterers, loose flooring, etc.). Piglets can be very destructive, especially as they grow and become stronger, such that their manipulation of pen objects could wreck the pens and producers could incur repair costs. In addition, the piglets’ welfare could be at risk if they ingest materials around the pen (such as rubber flooring pieces) or if they chew on a sharp corner of the metal feeder. Since piglets will engage in oral manipulation at the cost of their pen integrity and/or health, encouraging piglets to express their natural behaviors with a positive outlet (such as a burlap sheet) can improve their welfare by decreasing the occurrence of agonistic behaviors and resulting lesions, while satisfying their innate desire to explore their environment with their snout/ mouth.

Because the weaned piglets spent most of their time resting (Table 3), the scan behavior data were summarized as a proportion of time spent eating or drinking, being active, or interacting with the burlap, relative to their time spent resting for the purposes of the statistical analysis. There were no differences observed between treatments (*P *> 0.10).

**Table 3.** The average proportion of piglets across treatments performing state behaviors during observations did not differ between treatments comparing the effects of object enrichment (burlap provision) and preweaning mixing of litters (social enrichment)

	Proportion of piglets engaged in each behavior across treatments^1^
Burlap	3 ± 2%
Eating	8 ± 3%
Active	17 ± 6%
Resting	71 ± 9%

^1^Expressed as mean ± SD per experimental unit, per observation, per day, per piglet, 1N (*n* = 14 × 1 litter, not enriched), 1E (*n* = 15 × 1 litter, enriched), 2N (*n* = 16 × 2 litters, not enriched), 2E (*n* = 15 × 2 litters, enriched), 4N (*n* = 15 × 4 litters, not enriched), and 4E (*n* = 14 × 4 litters, enriched).

There was no observed difference between treatments for displacements at the feeder or for social behaviors (*P *> 0.10). Our results are consistent with those presented by Salazar et al. (2018) that social behaviors, positive interactions among pigs including play behaviors, were not different between socialized and non-socialized piglets within 1-wk of weaning; those authors only began to see differences in social behaviors 14 d after weaning. It is possible that changes in social behaviors were not observed during the trial as it trial ended at 7 d postweaning.

Similar to the results of Gardner et al. (2001), no difference in belly nosing was observed in our study. Although belly nosing is a behavior that is common in newly weaned pigs, it has been previously reported to be a poor indicator of stress in piglets (Gardner et al., 2001) because the causative effects behind the behavior are still unknown. It has been suggested that belly nosing may provide comfort through social contact for piglets performing the behavior however, it is weaning age that has been the strongest predictor of belly nosing, not food quality or quantity or other stressors according to previously published literature (Widowski et al., 2008).

### Lesion Scores

#### Effects of preweaning mixing (social enrichment)

The initial number of lesions per pig (within 24-h of weaning, transport, and sorting) was not significantly different between mixing factors. Consistent with other studies, piglet lesions were not affected by mixing until several days after weaning (day 4 postweaning: Kutzer et al., 2009; Fels et al., 2021) or 2 mo later (day 61 postweaning: D’Eath 2005). In contrast, the final lesion scores were influenced by both the mixing and enrichment factors. After 1 wk in the nursery, piglets from the mixed groups of four litters preweaning tended (*P* = 0.07) to have fewer lesions than the mixed groups of two or single-litter groups preweaning. Because establishing a hierarchy can take several days after mixing pigs (Tong et al., 2019), our lesion scoring schedule should have better captured any lesions resulting from aggression or fights establishing a hierarchy.

#### Effects of burlap provision (object enrichment)

The initial number of lesions per pig tended (*P *= 0.07) to be lower in enriched groups compared to non-enriched groups. Additionally, enriched groups tended (*P* = 0.10) to have fewer lesions per pig compared to the non-enriched groups after 1-wk postweaning. In comparison, Yang et al. (2018) did not observe enrichment effects on pig lesions; however, lesions were only scored 1 and 2 d after weaning in that study, whereas lesions were scored 1 and 7 d after weaning in this trial. Furthermore, our piglets had access to enrichment before and after weaning while the enrichment was only available until weaning in the previous study (Yang et al., 2018). This emphasizes the importance of having enrichment in the nursery to redirect agonistic behaviors from pen-mates to the enrichment device and is further supported by Oostindjer et al. (2011). It would be worth evaluating which timing of enrichment is most beneficial to piglets around weaning in a future study. In our study, piglets had access to burlap in the sow barn and the nursery to create familiarity between the two environments. Other studies either only provide the enrichment in the preweaning environment (Yang et al., 2018; Ko et al., 2020; Schmitt et al., 2020) or the postweaning environment (Ledergerber et al., 2015).

Across treatments, initial lesion scores were lower than final lesion scores. The increase in lesion scores over the week postweaning is to be expected because social hierarchies within groups of pigs are established approximately 1 wk after weaning, so the number of lesions would increase as the hierarchies are established through fighting and biting (Tong et al., 2019). Although, no significant difference was found in the calculated degree of change over time between initial and final lesion scores during the week after weaning (*P* > 0.10), both object and social enrichment tended to have a positive effect on the number of lesions per piglet 1 wk after weaning. This is of importance because reducing skin lesions in weaned pigs can also reduce the incidence and/or spread of common skin diseases like Exudative Epidermitis (greasy pig disease). Greasy pig disease is an endemic problem worldwide that is spread through contact between pigs; skin lesions increase its transmission by facilitating the entry and transfer of infectious bacteria (Park et al., 2013). Although Park et al. (2013) list many treatment options that are available through veterinary care, proactive and prevention measures through management practices such as mixing piglets preweaning and adding enrichment to their environment should be prioritized on-farm.

## Conclusion

Our treatment factors allowed adjacent litters to socialize preweaning (as groups of two or four litters) and/or interact with burlap as object enrichment pre- and postweaning. Overall, neither treatment affected initial piglet lesions or state behaviors (resting, eating/drinking, being active, using burlap) after weaning. However, the lesion scores measured postweaning indicated that when piglets had access to burlap, they tended to have fewer lesions initially (24 h after weaning) and 1-wk postweaning. Piglets mixed in larger groups (four litters mixed) preweaning also had fewer lesions 1-wk postweaning. Burlap use was observed the most in the pens of piglets that were mixed in groups of four litters, while in these same pens, there was less biting observed between piglets. There was less observed biting, displacements throughout the pen, and pen object manipulation in enriched pens of piglets compared to the non-enriched pens of piglets. Furthermore, enriched piglets were observed fighting less than non-enriched piglets. The frequency of agonistic behavior (i.e., biting) was reduced when piglets were provided with burlap and when they were mixed in groups of four litters preweaning which may, ultimately, improve piglet welfare at weaning.
